# Analysis of Factors Determining the Stiffness and Elasticity of Scars in Women After Cesarean Section—A Pilot Study

**DOI:** 10.3390/jcm15010264

**Published:** 2025-12-29

**Authors:** Katarzyna Strojek, Piotr Ożóg, Wojciech Smuczyński, Agnieszka Radzimińska, Magdalena Weber-Rajek, Hanna Styczyńska, Maciej Władysław Socha

**Affiliations:** 1 Department of Physiotherapy, Collegium Medicum in Bydgoszcz, Nicolaus Copernicus University in Toruń, 85-168 Bydgoszcz, Poland; 2 Department of Perinatology, Gynecology and Gynecological Oncology, Faculty of Health Sciences, Collegium Medicum in Bydgoszcz, Nicolaus Copernicus University in Toruń, 85-168 Bydgoszcz, Poland

**Keywords:** cesarean section, scar, stiffness, elasticity, myotonometry

## Abstract

**Background/Objectives:** Among women with a history of cesarean section (CS), scar abnormalities are observed in 20–32% of cases. The presence of a scar in the lower abdomen may cause pain, dysmenorrhea, dyspareunia, and postural dysfunction. The aim of the study was to conduct a feasibility study and secondary exploratory analysis of the factors determining scar stiffness and elasticity in women after CS. **Methods:** The study involved 30 women aged 26 to 45 who had undergone at least one CS no earlier than six months before the start of the study. The following feasibility endpoints were analyzed: recruitment rate, completion rate, protocol deviations, and device usability. Myotonometry was performed to quantify the stiffness and decrement (a parameter that inversely reflects tissue elasticity) of the CS scar. The correlation between stiffness and decrement and age, BMI, time since the las CS, and the number of CSs was assessed. **Results:** All predefined feasibility criteria were met. Recruitment exceeded the target rate (3.3 participants/week), with a high completion rate (90%). One minor protocol deviation occurred without impact on safety or data integrity. The MyotonPro device showed good usability, with no reported discomfort and successful completion of all measurements. Secondary exploratory correlation analyses suggested a tendency toward a negative correlation between BMI and the stiffness and decrement (indicating increased elasticity) across most scar regions. No consistent correlations were observed between age and the examined scar parameters. Exploratory analyses further indicated a probable positive correlation between time elapsed since the last CS and stiffness and decrement (indicating reduced elasticity), which was observed only in the central region of the scar. The number of cesarean section procedures showed sporadic, region-specific correlations with scar parameters, limited to selected measurement points. **Conclusions:** These findings suggest that the study design is feasible and acceptable. Future research protocols should also include a comparison with healthy skin in the scar area. Preliminary exploratory analyses suggest a potential influence of BMI and time since last CS on scar stiffness and elasticity. However, due to the limitations of the pilot study, these observations should be considered preliminary and hypothesis-generating and may be used to design future confirmatory studies.

## 1. Introduction

A cesarean section (CS) is a surgical procedure involving the delivery of the fetus via an abdominal incision (laparotomy) and a uterine incision (hysterotomy). It is typically performed when vaginal delivery poses increased risk to the mother or fetus—for example, due to complications during labor or maternal/fetal conditions preventing safe vaginal birth. It is estimated that CS is the most commonly performed and safest surgical procedure in the United States, with over 1 million cesarean deliveries annually. In 2022, more than 3.66 million births were recorded in the U.S., of which 32.2% were cesarean deliveries. The global rate of cesarean sections continues to increase, and in the United States, the number of non-medically indicated procedures remains a critical public health priority [1,2,3].

In recent years, a progressive increase in the incidence of cesarean deliveries (CDs) has been observed. The procedure has become the most frequently performed surgery in gynecology and obstetrics. In Polish hospitals, the percentage of births via CS rose from 13.8% in 1994 to 18.1% in 1999, 28.8% in 2006, 37% in 2021, 43% in 2015, and 45% in both 2019 and 2020. By 2021 and 2022, the rates had further increased to 37% and 48%, respectively. The highest regional rate—58% in 2022—was reported in the Podlaskie Voivodeship. The increasing prevalence of CS is mainly attributed to scheduled repeat cesarean deliveries (due to the risk of uterine rupture) and a decline in spontaneous breech births. While obstetricians and midwives actively promote vaginal delivery, experts predict that a significant reduction in cesarean rates is unlikely over the next decade [2].

The number of women with a history of cesarean section is increasing. These patients often become pregnant again and face a risk of cesarean scar dehiscence or uterine rupture. These complications, which may be asymptomatic or paucisymptomatic, pose serious risks to both maternal and fetal health and are often difficult to diagnose. CS is a major surgical procedure associated with risks of infection and hemorrhage, and the recovery period is typically longer than after vaginal delivery [1].

Among women with a history of at least one CS, cesarean scar abnormalities are observed in 20–32% of cases. The presence of a scar in the lower abdomen may cause pain, dysmenorrhea, dyspareunia, and postural dysfunction. Women after CS more frequently experience pelvic floor dysfunction, pelvic pain syndromes, or low back pain. These conditions negatively affect the patient’s quality of life—physically, psychologically, and socially—resulting in diminished self-esteem, body image dissatisfaction, and increased healthcare costs. Many women expect the cesarean scar to be barely visible and concealed by underwear; however, this is not always achieved [2].

A cesarean scar pregnancy (CSP) is a very rare but potentially dangerous complication.

The effectiveness of pelvic floor dysfunction treatment depends not only on the choice of therapeutic techniques and interventions but also on the accurate diagnosis and timely initiation of therapy. The integration of the scar with adjacent soft tissues (fascia and muscles) is a fundamental element of postoperative rehabilitation and essential for full recovery. Patients with pelvic dysfunction often initially attempt to manage symptoms independently; however, as symptoms progress, they tend to withdraw from physical, professional, and social activities or even become socially isolated. In recent years, there has been growing interest among physiotherapists in scar therapy and a dynamic development of urogynecological and obstetric physiotherapy. The primary aim of physiotherapeutic manual techniques is to remodel the rough and uneven structure of scars and integrate them with surrounding tissues. Integrating the scar into the fascial network reduces the risk of dehiscence, particularly in scars with compromised tensile strength [4,5,6,7,8,9,10].

In clinical practice, scar diagnostics involves both subjective and objective tools. Standardized scar assessment scales include the Vancouver Scar Scale (VSS), Manchester Scar Scale (MSS), Patient and Observer Scar Assessment Scale (POSAS), and Stony Brook Scar Evaluation Scale (SBSES). Objective tools are used to assess pain in and around the scar (e.g., a dolorimeter) and to quantify scar stiffness, hardness, and elasticity using devices such as a cutometer, dermal torque meter, adheremeter, or myotonometer. Scar assessment may also be supplemented by ultrasonographic examination of skin layers and scar tissue. These tools are useful for both initial assessment and monitoring therapeutic progress [11,12,13].

## 2. Study Objective

The study aimed to assess the feasibility of the research procedures, tools, and methods in order to refine them before the main study. Additionally, a secondary exploratory analysis was performed to investigate the relationships between selected factors—age, BMI, time since the last CS, and the number of cesarean sections—and cesarean scar properties, including stiffness and elasticity.

## 3. Methods

### 3.1. Study Design

The protocol of this study was formally validated and registered on ClinicalTrials.gov under the registration number NCT06446258. The research was conducted at the Department of Physiotherapy, Collegium Medicum in Bydgoszcz, Nicolaus Copernicus University in Toruń, between September 2024 and February 2025. Information about the tests was made available at the Department of Physiotherapy, Collegium Medicum in Bydgoszcz (posters in display cases, information on Facebook, and the university website).

Participants were recruited based on an original interview questionnaire and a functional examination, according to the following inclusion and exclusion criteria.

Inclusion criteria:

- Women aged 18–45 years;
- A history of at least one cesarean section performed no less than six months prior to the study;
- No physiotherapeutic treatment of the scar within the last six months.

Exclusion criteria:

- Age over 45 years;
- Presence of abdominal mesh;
- History of abdominal plastic surgery;
- Nephrectomy, hysterectomy, or cystectomy;
- Uterine fibroids or stoma;
- Less than six months since the last cesarean section;
- Presence of neurological symptoms (based on a functional neurological examination, including Slump Test, Babinski sign, clonus reflex testing, Straight Leg Raise Test, Bowstring Test, femoral nerve tension tests in prone and side-lying positions, deep tendon reflexes—patellar and Achilles, superficial sensation, and manual muscle testing for key muscles innervated from the lumbosacral spine);
- Positive findings in pain provocation tests, including axial spinal compression and maximum intervertebral foramen compression in the lumbosacral region;
- History of spinal trauma or surgery;
- Vertical cesarean scar;
- Participation in scar-related physiotherapy in the last 6 months;
- The presence of the following comorbidities: oncological, endocrinological (e.g., diabetes), osteoporotic, gastrointestinal, cardiovascular, rheumatic, psychiatric, or gynecological conditions;
- Current pregnancy.

All participants received a written information sheet regarding the study procedures and provided written informed consent prior to participation.

### 3.2. Participants

A total of 30 women were included in the study, aged between 26 and 47 years (inclusion criteria protocol deviation), with a mean age of 37 years. The mean body mass index (BMI) was 25.7, with values ranging from a minimum of 19.3 to a maximum of 44.9. The time elapsed since the last cesarean section was also analyzed. On average, it was 61 months, ranging from a minimum of 6 months to a maximum of 264 months.

Descriptive statistics for key variables—including age, weight, height, BMI, and time since last cesarean section—are presented in Table 1. Frequencies related to the number of cesarean sections per participant are shown in Table 2.

According to the Shapiro–Wilk normality test, age and height conformed to a normal distribution (*p* > 0.05), whereas the other variables deviated significantly (*p* < 0.05).

### 3.3. Feasibility Study

To perform the feasibility analysis, the following endpoints were analyzed: recruitment rate per time, completion rate, protocol deviations, and device usability. The following formulas were used to calculate the recruitment rate per time and completion rate:

$$Recruitment\;rate=\frac{number\;of\;participtans\;recruited}{recruitment\;time\;\left(weeks\right)}$$

$$Completion\;rate=\frac{number\;of\;participtans\;who\;completed\;the\;study}{number\;of\;participants\;who\;started\;the\;study}\times 100\%$$

▪ According to the CONSORT Extension for Pilot and Feasibility Trials, feasibility thresholds were defined as a recruitment rate of at least 2 participants per week and a completion rate of at least 80%.
▪ Protocol deviations and device usability are presented descriptively (qualitatively).

Protocol deviations were defined as any instance in which a study procedure or eligibility criterion was not followed as specified in the study protocol. All deviations were recorded, classified as major or minor, and included in the study results to inform future modifications.

Protocol deviations were classified as follows:

- Major deviation (critical deviation): Deviations that could compromise participant safety or data integrity, such as omission of critical study procedures or enrollment of participants who did not meet inclusion/exclusion criteria, except for a single deviation from the age criterion.
- Minor deviation (non-critical deviation): A single enrollment outside the age criterion, delays in the visit schedule, or minor deviations in measurement procedures that do not impact data safety or data quality.

The feasibility criterion for protocol deviations was defined as the absence of major deviations that could compromise overall study procedures or data integrity. Minor deviations, including a single enrollment outside the age criterion, were recorded for protocol refinement but did not affect the assessment of feasibility.

The feasibility of using the MyotonPro device (Myoton AS, Tallinn, Estonia) in the study was assessed based on the following predefined criteria (Table 3):

### 3.4. Myotonometry Measurements

A custom-designed questionnaire was used in this study to collect demographic data, including age, weight, and height of the participants. The questionnaire also included questions regarding the number and timing of previous deliveries, distinguishing between vaginal births and cesarean sections, as well as a history of other surgical procedures and comorbid conditions.

Each participant underwent myotonometric assessment to quantitatively evaluate tissue tension, as well as biomechanical and viscoelastic properties of soft tissues in the area of the cesarean scar. For this purpose, the MyotonPro device (Myoton SA, Tallinn, Estonia) was used. This tool is recognized as a reliable instrument for the objective measurement of viscoelastic properties of scar tissue after cesarean section and of non-scarred skin. The inter-rater and intra-rater reliability of these measurements is considered good to excellent (intraclass correlation coefficients [ICCs] 0.99–1.00 and 0.87–0.98, respectively) [13].

The measurement protocol involved applying the probe of the myotonometer perpendicularly to the surface of the scar at three distinct locations: the beginning, the middle, and the end of the scar. Once proper contact was established, the device delivered a brief mechanical impulse (15 ms) with low force, without inducing reflexive abdominal muscle contractions. This impulse generated mechanical oscillations in the tested area. These oscillations were then automatically processed by the device’s accelerometric sensor system to calculate specific tissue parameters [14].

The following two parameters were analyzed:

- Stiffness (N/m): Indicates the tissue’s resistance to external mechanical forces and reflects overall tissue stiffness.
- Decrement (logarithmic decrement): A parameter that inversely reflects tissue elasticity, i.e., the ability to return to its original shape after deformation. A higher decrement value corresponds to lower elasticity.

During the myotonometric examination, each patient was positioned supine, with a roller under her knees. Each patient received instructions from the researcher regarding the exact moment of the myotonometric measurement. The researcher asked the patient to inhale freely, then exhale freely, and the measurement was taken at the end of each patient’s expiratory phase. The order of measurement for each patient was the same: first, the beginning of the scar (right side), then the center of the scar (umbilical line), and finally, the end of the scar (left side). The authors did not know whether the physician performing the cesarean section was right- or left-handed, so we were unable to determine the actual beginning and end of the scar. The authors therefore assumed that most gynecologists were right-handed, and we considered the right side of the scar as the beginning of the scar.

### 3.5. Statistical Analyses

The statistical analysis of the data collected during the study was performed using JASP (Version 0.95.4). To analyze the variables associated with factors determining scar stiffness and elasticity in women after cesarean section, the following statistical tests were applied:

- Spearman’s rank correlation test—to assess the strength and direction of the monotonic association between two variables. This method is suitable for continuous variables with non-normal distribution, as well as for ordinal data, and is relatively robust to outliers (Spearman’s rho).
- Shapiro–Wilk test—to evaluate the normality of distribution. A significant result (*p* < 0.05) indicates that the data deviate significantly from a normal distribution.
- Mann–Whitney U test—to compare the distributions of two independent groups. A significant result (*p* < 0.05) suggests statistically significant differences. This is the non-parametric equivalent of the independent samples *t*-test.
- Wilcoxon signed-rank test—to compare two related measurements (e.g., before and after treatment) in the same group. A significant result (*p* < 0.05) indicates differences between the paired conditions. This is the non-parametric equivalent of the paired samples *t*-test.

## 4. Results

### 4.1. Feasibility Study

#### 4.1.1. Recruitment Rate

During the 9-week recruitment period, 30 participants were enrolled, corresponding to a recruitment rate of 3.3 participants per week. This rate exceeded the predefined feasibility criterion of two participants per week.

#### 4.1.2. Completion Rate

Thirty participants were initially enrolled in the study. Of these, 27 completed all study stages, resulting in a completion rate of 90%, which exceeded the predefined feasibility threshold of 80%. Three participants dropped out (dropout rate of 10%). To obtain the planned number of 30 complete datasets for secondary exploratory analysis, three additional participants were subsequently recruited and completed the study according to the protocol.

#### 4.1.3. Protocol Deviations

One participant was enrolled despite not meeting the age criterion (47 years, with a protocol cutoff of ≤45 years). This deviation was identified after study procedures had begun. As this was the only protocol deviation and it did not affect participant safety or data integrity, it was classified as a minor deviation and did not influence the feasibility assessment.

#### 4.1.4. Device Usability

The MyotonPro^®^ device used in the study met all predefined feasibility criteria for device usability. Patients reported no discomfort during the examination, and the researchers noted no major errors in the device’s operation. Using the device was simple and intuitive. In some cases, it was difficult to properly place the myotonometer’s measuring tip on the scar tissue—only proper positioning resulted in the device’s green LED illuminating, allowing for measurement. However, this could be due to the variability of scar structure and the researchers’ inexperience in performing the measurements. Ultimately, all measurements were completed successfully, and despite minor delays, the patients did not complain about excessively long measurement times. The examination procedure, including the breathing commands during the measurement, was fully understandable to the patients and did not require repetition.

### 4.2. Secondary Exploratory Analysis of the Correlation Between Age, BMI, and Time Since the Last Cesarean Section and Scar Stiffness and Elasticity

Due to the small sample size, the presence of outliers, and the non-normal distribution of variables, Spearman’s rank correlation coefficient (rho) was used to assess monotonic relationships between variables. The values provided in the tables include Spearman’s rho and associated *p*-values. Statistically significant correlations are marked with asterisks (see table footnotes). The magnitude and sign of the rho coefficient indicate the strength and direction of the relationship.

**Figure 1:**
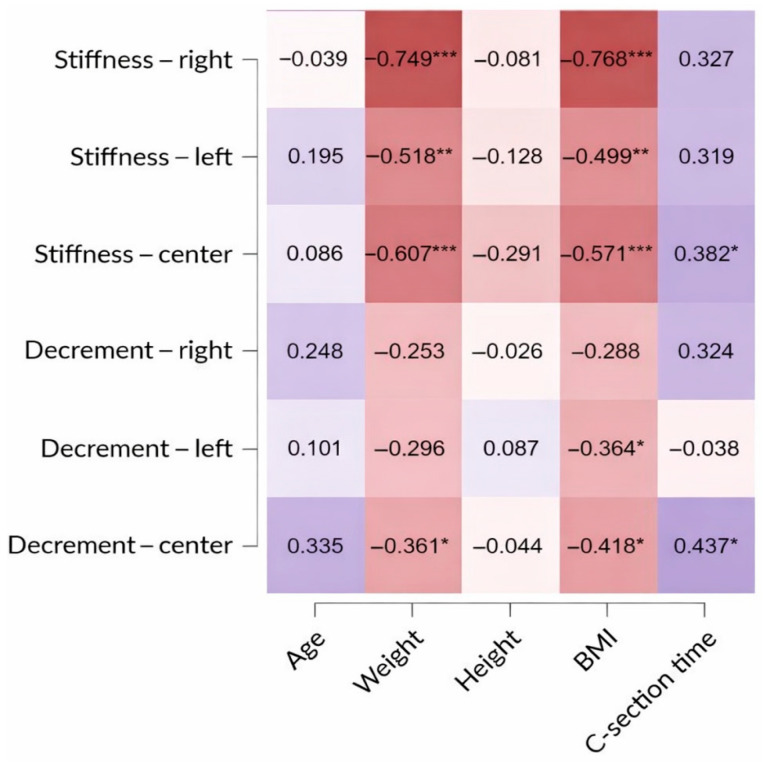
Heatmap of Spearman’s rho correlations. The direction of the correlation is indicated by color (blue = positive, red = negative); color intensity represents correlation strength (* *p* < 0.05, ** *p* < 0.01, *** *p* < 0.001.

According to the data presented in Table 4 and Figure 1, exploratory analyses suggested a negative correlation between BMI and scar stiffness at all three measured points (right side, left side, and central area of the scar). For decrement, a similar correlation was observed at two out of the three locations (left side and center of the scar), which may indicate increased elasticity. Partial correlations for BMI were also analyzed (Table 5). We examined whether the BMI correlations persisted after accounting for time since the last cesarean section and age.

As shown in Table 4, the associations with BMI persisted after accounting for C-section time and age.

Exploratory analyses did not reveal any significant associations between participants’ age and scar stiffness or decrement at any measurement point. However, a secondary exploratory analysis suggested a possible positive correlation between the time elapsed since the last cesarean section and stiffness and decrement (indicating reduced elasticity), observed only in the central region of the scar.

### 4.3. Scar Stiffness and Elasticity in Relation to the Number of Cesarean Sections

The number of cesarean sections (CSs) performed on each participant was used to divide the group into three subgroups: 1 CS, 2 CS, and 3 CS. However, since only one participant had undergone three cesarean sections, the 2 CS and 3 CS groups were merged, resulting in two comparison groups: 1 CS and >1 CS (i.e., 2 or more cesarean sections) (Table 6). Baseline MyotonPro^®^ measurements of scar stiffness and elasticity were compared between these two groups. Due to the small sample size and the fact that the Shapiro–Wilk test (Table 7) indicated non-normal distribution in most variables (*p* < 0.05), the non-parametric Mann–Whitney U test was selected to assess between-group differences.

**Figure 2:**
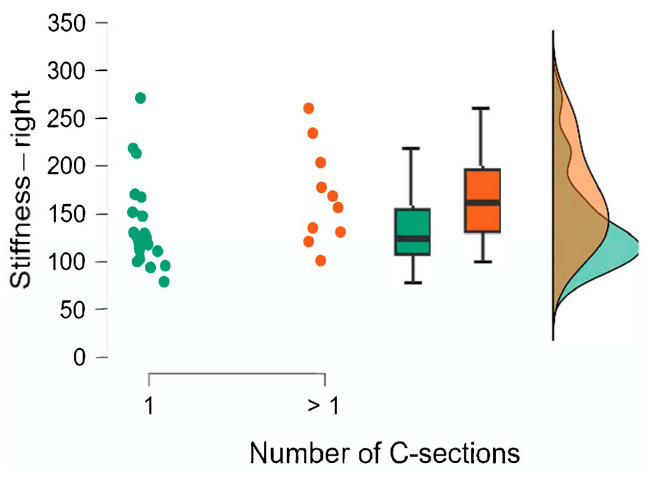
Number of C-sections. Stiffness—right.

**Figure 3:**
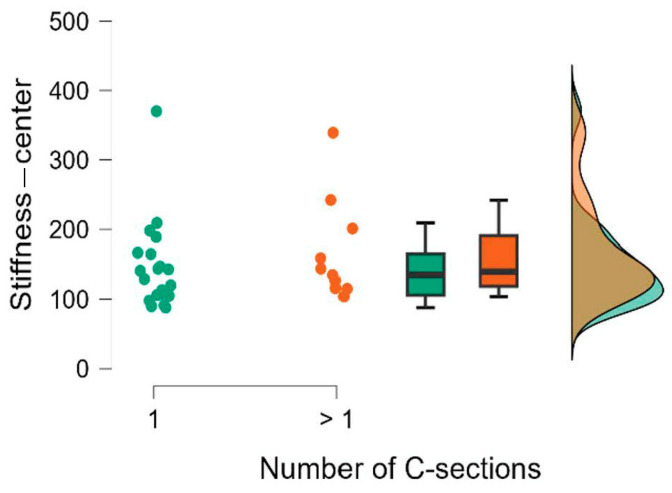
Number of C-sections. Stiffness—center.

**Figure 4:**
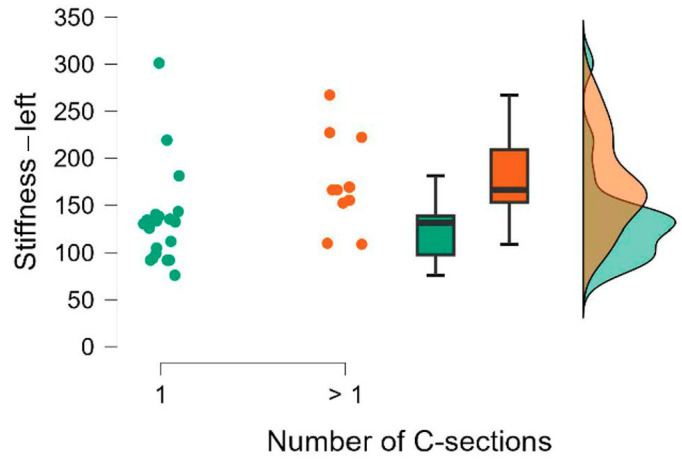
Number of C-sections. Stiffness—left.

**Figure 5:**
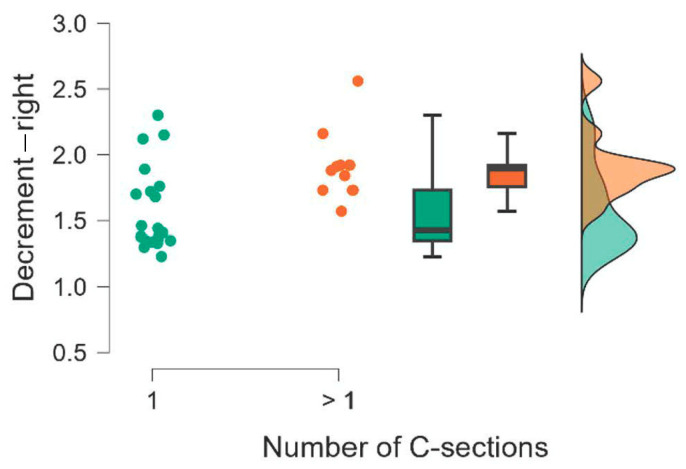
Number of C-sections. Decrement—right.

**Figure 6:**
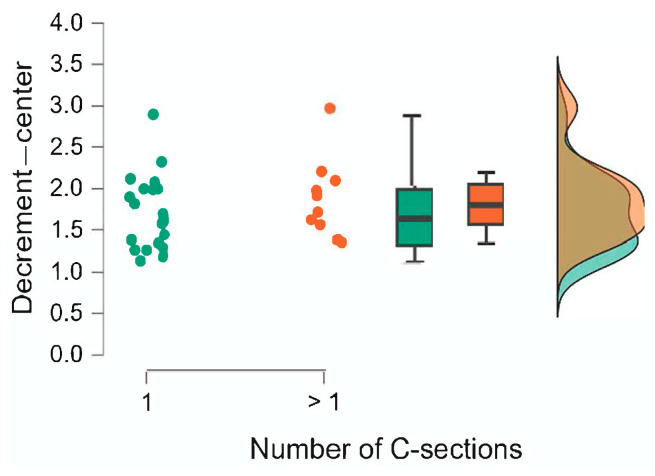
Number of C-sections. Decrement—center.

**Figure 7:**
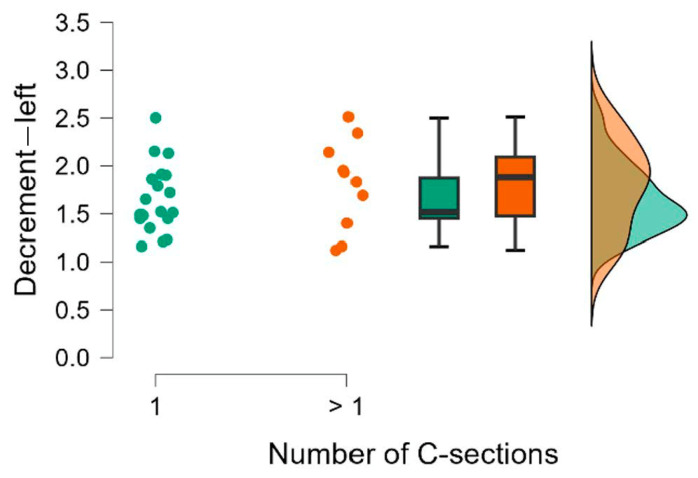
Number of C-sections. Decrement—left.

The results of the Mann–Whitney U test are presented in Table 8. We also calculated the rank-biserial correlation effect size r, which is interpreted as small for 0.11 ≤ r < 0.28, medium for 0.28 ≤ r < 0.43, and large for r ≥ 0.43 [15]. The comparison of the groups is also illustrated using raincloud plots (Figure 2, Figure 3, Figure 4, Figure 5, Figure 6 and Figure 7).

## 5. Discussion

A scar forms as a result of skin injury, which may be caused by mechanical trauma, burns, dermatological conditions, biological or chemical agents, or surgical procedures. Proper wound healing typically results in a scar that is aesthetically acceptable—flat, pale, or elastic—and confined to the original injury site. Several factors, including age, anatomical location, ethnicity, and type of injury, can increase the risk of pathological scar formation. Pathological scars such as hypertrophic scars, keloids, and atrophic scars remain a major challenge in tissue repair, with a reported prevalence ranging from 4.5% to 16% [13].

The main aim of this study was to conduct a pilot feasibility assessment to refine research procedures, tools, and methods prior to a full-scale trial. The sample size of 30 participants was chosen in line with recommendations for pilot studies, which do not require formal sample size calculations but rather suggest tailoring the number of participants to the study group and selected feasibility indicators [16,17,18]. Previous pilot studies involving women after cesarean section reported recruitment ranges between 10 and 32 participants [19,20,21,22], supporting the appropriateness of our sample for evaluating recruitment, completion, and measurement feasibility.

Recruitment proceeded at a rate of 3.3 participants per week, exceeding the predefined target. Completion rate was high (90%), with only three participants discontinuing for reasons unrelated to the protocol (illness, pregnancy, or missed appointments). One minor protocol deviation occurred (inclusion of a participant slightly above the age cutoff), which did not affect safety or data integrity. The MyotonPro device was feasible and well-tolerated, with all planned measurements successfully completed. Occasional difficulties in positioning the device on scar tissue were minor and did not compromise data collection. Overall, the study design and measurement procedures were practical, acceptable to participants, and suitable for scaling to a larger trial.

Secondary analyses explored relationships between participant characteristics (BMI, age, time since the last cesarean section, and number of cesarean procedures) and scar biomechanical properties. Higher BMI may be associated with reduced stiffness and increased elasticity of the transverse cesarean scar. This may reflect the influence of subcutaneous adipose tissue on mechanical measurements [23,24]. Moreover, no prior studies have investigated the influence of anthropometric factors (e.g., height, weight, and BMI) on scar tissue parameters. This makes direct comparison with previous research challenging. However, our findings can be discussed in the context of other studies that focused on therapeutic interventions involving cesarean scars. Prior studies support the reliability of MyotonPro measurements. For instance, Gilbert et al. reported higher stiffness in cesarean scars compared to adjacent skin but no significant differences in elasticity and demonstrated excellent intra- and inter-rater reliability [13]. Therapeutic interventions, such as manual therapy, have been shown to reduce stiffness and improve elasticity, supporting the potential clinical utility of myotonometric assessment in monitoring scar tissue [21].

No significant associations with age were observed, likely due to the small number of younger participants. Similar findings were reported by Rosicka et al., who found no differences in skin elasticity at three anatomical points in a relatively homogeneous group of women aged 19–25 years [25]. Earlier studies, however, reported that skin elasticity is age-dependent, with more pronounced variation when comparing younger and older populations [26,27].

Time since the last cesarean section suggested the presence of a positive correlation with stiffness and decrement but only in the central region of the scar, potentially reflecting progressive fibrosis over time. A study by Wassermann et al. confirmed that the number of years since cesarean section was the only significant predictor of group differences (*p* = 0.011; partial η^2^ = 0.438), while other measures (pain threshold, scar mobility, Oswestry Disability Index, GROC, and pain NRS) showed no significant associations [28].

Finally, in this pilot study, region-specific associations were observed between the number of cesarean procedures and scar properties, but these were inconsistent, precluding definitive conclusions. These findings are exploratory and should be interpreted with caution.

Our pilot study results suggest that myotonometric assessment of cesarean scars is feasible and may provide objective, region-specific information. Early identification of increased stiffness could inform preventive or therapeutic interventions, such as targeted manual therapy, particularly in the central scar region and in patients with a longer time since surgery. Findings also highlight the importance of individualized assessment rather than relying solely on BMI or other general risk factors when evaluating scar quality. This knowledge can help reassure patients and guide attention toward other aspects of scar care and overall healing.

To optimize recruitment and feasibility in a larger trial, expanding recruitment channels within a multicenter collaboration is recommended, including gynecology and obstetrics outpatient clinics or hospital departments. This approach could increase participant diversity and accelerate enrollment, facilitating more robust future investigations. Future studies should consider increasing sample size and age stratification to better assess age-related effects. Including a comparison with healthy skin adjacent to the scar would also significantly increase the objectivity and interpretability of the obtained results. Finally, future studies may include additional screening steps to prevent minor protocol deviations, such as the inclusion of participants above the age cutoff.

This pilot study has several limitations. The sample size was small, which may limit the generalizability of the findings and the statistical power to detect associations. Additionally, stiffness and elasticity measurements were not performed on non-scarred skin adjacent to the cesarean scar, which would have provided a more objective reference for comparison. Future larger-scale studies should include myotonometric assessments of both scarred and adjacent healthy skin to enhance the interpretability and clinical relevance of the results.

## 6. Conclusions

These findings suggest that the study design is feasible and acceptable. Future research protocols should also include a comparison with healthy skin in the scar area.

Preliminary exploratory analyses suggest a potential influence of BMI and time since last CS on scar parameters. Higher BMI may be associated with reduced stiffness and increased elasticity of the transverse cesarean scar. The time elapsed since the last CS may contribute to localized increases in stiffness and a reduction in elasticity, particularly in the central region of the scar. However, due to the limitations of the pilot study, these observations should be considered preliminary and hypothesis-generating and may be used to design future confirmatory studies.

## Figures and Tables

**Table 1:** Descriptive statistics: age, weight, height, BMI, and time since last CS.

Variable	Median	Mean	Standard Deviation	IQR	Shapiro–Wilk	Shapiro–Wilk *p*-Value	Minimum	Maximum
Age	36.500	37.167	5.453	8.250	0.959	0.296	26.000	47.000
Weight	71.000	72.567	16.158	15.500	0.858	<0.001	55.000	130.000
Height [m]	1.680	1.679	0.058	0.060	0.968	0.493	1.560	1.830
BMI	24.484	25.700	5.279	6.120	0.860	0.001	19.377	44.983
Time since last CS [months]	33.500	61.967	64.325	75.000	0.805	<0.001	6.000	264.000

BMI—body mass index; CS—cesarean section.

**Table 2:** Frequency distribution: number of CS procedures.

Number of CS	Frequency	Percent
1	20	66.667
2	9	30.000
3	1	3.333
Missing data	0	0.000
Total	30	100.000

CS—cesarean section.

**Table 3:** Device usability feasibility criteria.

Feasibility Criterion	Definition/Threshold	Notes
Successful completion of measurements	≥95% of planned measurements yield valid data	*Ensures the device can be used to collect reliable data for all participants.*
Patient safety and comfort	No reported discomfort, pain, or adverse events	*Participant well-being is prioritized; minor transient discomfort may be acceptable if rare.*
Absence of critical operational errors	No device malfunctions or user errors prevent measurement completion	*Minor operational difficulties that do not affect measurement success are acceptable.*
Measurement duration	Measurement time within the planned schedule, with minor delays allowed in <10% of measurements	*Ensures practicality in clinical or research settings.*
Ease of device operation by researchers	Device can be operated correctly without extensive training	*Minor difficulties,* e.g., *positioning the measuring tip on scar tissue, are acceptable if they do not prevent a valid measurement.*
Participant’s understanding of the procedure	≥95% of participants understand instructions without repeated explanations	*Ensures clarity of procedure and participant compliance.*

**Table 4:** Spearman’s correlations with bootstrap 95% CIs (from 1000 bootstraps).

Variable		Age	Weight	Height	BMI	C-Section Time
Stiffness—right	Spearman’s rho	−0.039	−0.749 ***	−0.081	−0.768 ***	0.327
	*p*-value	8.367 × 10^−1^	1.920 × 10^−6^	6.692 × 10^−1^	7.158 × 10^−7^	7.731 × 10^−2^
	Lower 95% CI	−0.479	−0.864	−0.454	−0.872	−0.105
	Upper 95% CI	0.395	−0.554	0.295	−0.604	0.698
Stiffness—left	Spearman’s rho	0.195	−0.518 **	−0.128	−0.499 **	0.319
	*p*-value	3.009 × 10^−1^	3.354 × 10^−3^	5.014 × 10^−1^	4.989 × 10^−3^	8.533 × 10^−2^
	Lower 95% CI	−0.208	−0.727	−0.494	−0.710	−0.098
	Upper 95% CI	0.575	−0.189	0.224	−0.176	0.678
Stiffness—center	Spearman’s rho	0.086	−0.607 ***	−0.291	−0.571 ***	0.382 *
	*p*-value	6.506 × 10^−1^	3.705 × 10^−4^	1.189 × 10^−1^	9.724 × 10^−4^	3.745 × 10^−2^
	Lower 95% CI	−0.345	−0.778	−0.586	−0.800	−0.061
	Upper 95% CI	0.492	−0.333	0.082	−0.280	0.744
Decrement—right	Spearman’s rho	0.248	−0.253	−0.026	−0.288	0.324
	*p*-value	1.855 × 10^−1^	1.770 × 10^−1^	8.927 × 10^−1^	1.227 × 10^−1^	8.101 × 10^−2^
	Lower 95% CI	−0.135	−0.584	−0.406	−0.590	−0.059
	Upper 95% CI	0.601	0.145	0.361	0.097	0.619
Decrement—left	Spearman’s rho	0.101	−0.296	0.087	−0.364 *	−0.038
	*p*-value	5.943 × 10^−1^	1.121 × 10^−1^	6.482 × 10^−1^	4.828 × 10^−2^	8.421 × 10^−1^
	Lower 95% CI	−0.281	−0.643	−0.324	−0.675	−0.456
	Upper 95% CI	0.472	0.090	0.442	−0.019	0.370
Decrement—center	Spearman’s rho	0.335	−0.361 *	−0.044	−0.418 *	0.437 *
	*p*-value	7.065 × 10^−2^	4.998 × 10^−2^	8.177 × 10^−1^	2.165 × 10^−2^	1.577 × 10^−2^
	Lower 95% CI	−0.037	−0.622	−0.416	−0.669	0.080
	Upper 95% CI	0.707	0.013	0.313	−0.094	0.721

* *p* < 0.05, ** *p* < 0.01, *** *p* < 0.001.

**Table 5:** BMI, partial out: C-section time and age.

Variable		Stiffness—Right	Stiffness—Left	Stiffness—Center	Decrement—Right	Decrement—Left	Decrement—Center
BMI	Spearman’s rho	−0.738 ***	−0.509 **	−0.534 **	−0.291	−0.457 *	−0.450 *
	*p*-value	7.427 × 10^−6^	5.657 × 10^−3^	3.451 × 10^−3^	1.333 × 10^−1^	1.441 × 10^−2^	1.638 × 10^−2^
	Lower 95% CI	−0.873	−0.726	−0.780	−0.627	−0.719	−0.744
	Upper 95% CI	−0.512	−0.155	−0.178	0.097	−0.160	−0.139

* *p* < 0.05, ** *p* < 0.01, *** *p* < 0.001.

**Table 6:** Descriptive statistics: number of cesarean sections vs. scar stiffness and elasticity.

	Number of C-Sections	N	Median	Mean	Std. Deviation	IQR	Minimum	Maximum
Stiffness—right	1	20	125.500	138.850	48.324	46.000	79.000	271.000
Stiffness—right	>1	10	162.000	168.600	51.015	64.500	101.000	260.000
Stiffness—left	1	20	131.000	133.700	51.476	40.750	76.000	301.000
Stiffness—left	>1	10	166.000	174.300	50.640	56.000	109.000	267.000
Stiffness—center	1	20	135.000	146.000	64.164	59.750	88.000	370.000
Stiffness—center	>1	10	139.500	168.200	73.667	71.750	104.000	339.000
Decrement—right	1	20	1.425	1.583	0.318	0.380	1.230	2.300
Decrement—right	>1	10	1.895	1.922	0.273	0.163	1.570	2.560
Decrement—left	1	20	1.515	1.647	0.348	0.420	1.160	2.500
Decrement—left	>1	10	1.880	1.807	0.471	0.620	1.120	2.510

**Table 7:** Test of normality (Shapiro–Wilk).

Residuals	W	*p*
Stiffness—right	0.910	0.015
Stiffness—left	0.867	0.001
Stiffness—center	0.792	<0.001
Decrement—right	0.871	0.002
Decrement—left	0.980	0.829
Decrement—center	0.915	0.020

Note. Significant results suggest a deviation from normality.

**Table 8:** Comparison of the 1 CS and >1 CS groups (Mann–Whitney U test).

					95% CI for Rank-Biserial Correlation	
	U	*p*	Rank-Biserial Correlation	SE Rank-Biserial Correlation	Lower	Upper
Stiffness—right	58.000	6.730 × 10^−2^	0.420	0.223	0.002	0.713
Stiffness—left	44.000	1.456 × 10^−2^	0.560	0.223	0.185	0.793
Stiffness—center	78.500	3.555 × 10^−1^	0.215	0.223	−0.223	0.581
Decrement—right	36.000	5.197 × 10^−3^	0.640	0.223	0.303	0.835
Decrement—left	78.500	3.554 × 10^−1^	0.215	0.223	−0.223	0.581
Decrement—center	77.500	3.329 × 10^−1^	0.225	0.223	−0.213	0.588

## Data Availability

The datasets generated during and/or analyzed during the current study are available from the corresponding author on reasonable request.
